# Phylogeny and Evolutionary Timescale of Muscidae (Diptera: Calyptratae) Inferred from Mitochondrial Genomes

**DOI:** 10.3390/insects14030286

**Published:** 2023-03-15

**Authors:** Xin Li, Xiaodong Cai, Shuangmei Ding, Liang Wang, Wenliang Li, Xiaoyan Liu, Chuntian Zhang, Ding Yang

**Affiliations:** 1 College of Plant Protection, China Agricultural University, Beijing 100193, China; 2 The Institute of Scientific and Technical Research on Archives, National Archives Administration of China, Beijing 100050, China; 3 College of Horticulture and Plant Protection, Henan University of Science and Technology, Luoyang 471023, China; 4 College of Plant Science and Technology, Huazhong Agricultural University, Wuhan 430070, China; 5 College of Life Science, Shenyang Normal University, Shenyang 110034, China

**Keywords:** Azeliinae, Stomoxyinae, Reinwardtiinae, divergence time

## Abstract

**Simple Summary:**

Reconstruction of the phylogenetic relationship within Muscidae inferred by 43 mitochondrial genomes has revealed the split of the family into three major groups. The best phylogenetic tree recovers the monophyly of all subfamilies except Mydaeinae. Genus *Helina* Robineau-Desvoidy, 1830 was synonymized with *Phaonia* Robineau-Desvoidy, 1830. We prefer the subfamily status of Azeliinae and Reinwardtiinae and separate Stomoxyinae from Muscinae. Muscidae diverged in the early Eocene (51.59 Ma) and most subfamilies diverged at around 41 Ma.

**Abstract:**

House flies (Muscidae) comprise the most species-rich family of the muscoid grade with over 5000 described species worldwide, and they are abundant in various terrestrial and aquatic ecosystems. The high number of species, varied appearances, complex feeding habits, and wide distributions have hindered researchers from understanding their phylogeny and evolutionary history. Here, we newly sequenced fifteen mitochondrial genomes and reconstructed the phylogenetic relationships and divergence time among eight subfamilies of Muscidae (Diptera). The best phylogenetic tree, which was inferred by IQ-Tree, recovered the monophyly for seven out of eight subfamilies (except for Mydaeinae). Based on phylogenetic analyses and morphological characteristics, we prefer the subfamily status of Azeliinae and Reinwardtiinae, and separate Stomoxyinae from Muscinae. Genus *Helina* Robineau-Desvoidy, 1830 was synonymized with *Phaonia* Robineau-Desvoidy, 1830. The divergence time estimation indicated Muscidae originated at 51.59 Ma (early Eocene). Most subfamilies had originated around 41 Ma. We provided a mtgenomic viewpoint on the phylogenetic relationships and divergence time estimation of Muscidae.

## 1. Introduction

The Muscidae family, commonly known as house flies, are one of the most prominent dipteran families. They are recognized as essential for pollination, recycling, and as a food resource for birds, spiders, and many other animals. As one of the most species-rich families among the muscoid grade, they contain 5579 described species belonging to 207 genera worldwide [1]. They can be found in a variety of terrestrial and aquatic habitats, spanning most continents of the world, i.e., from arctic to tropical regions, with only limited exceptions for the most arid environments (e.g., *Synthesiomyia* Brauer *et* Bergenstamm, 1893; *Graphomya* Robineau-Desvoidy, 1830; *Limnophora* Robineau-Desvoidy, 1830; *Lispe* Latreille, 1796; etc.) [2,3,4]. Adult muscid flies include predators, bloodsuckers, and detritivores. Many species are also anthophilous, while larvae are saprophagous, coprophagous, predaceous, or more rarely phytophagous [5]. They also feed on numerous exudates from plants or animals [6]. The association between humans and Muscidae comprises agricultural, medical, and veterinary aspects [3,7]. There are many reports about Muscidae in forensic entomology, disease transmission, and livestock parasitism [2,4,6,8]. About 200 Muscidae species are known to visit human bodies or animal carrion [4]. Different species represent various hazards to livestock and humans, such as sucking blood, parasitism, and spreading of disease [6]. Additionally, muscid flies can be used as laboratory animals to test the toxic effects of pharmaceuticals and nanomaterials [9,10].

The small body size (2–8 mm) and significant variation in appearance make identification of many species within Muscidae difficult. The subfamily classification in Muscidae, as proposed by various authors, has been incongruent for a long time. Skidmore (1985) presented a fundamental ten-subfamily classification system mainly based on characteristics of the immature and mature stages (Achanthipterinae, Atherigoninae, Azeliinae, Coenosiinae, Eginiinae, Reinwardtiinae, Stomoxyinae, Muscinae, Mydaeinae, and Phaoniinae) [3]. Carvalho (1989) proposed a seven-subfamily classification based on adult characteristics (Achanthipterinae, Atherigoninae, Azeliinae, Coenosiinae, Muscinae, Mydaeinae, and Phaoniinae) [11]. Comparable to Skidmore (1985), who treated Stomoxyini, Reinwardtiini, and Eginiini as subfamilies [12], Xue and Chao (1998) divided Muscidae species in China into ten subfamilies. Carvalho et al. (2005) suggested a seven-subfamily classification for the Neotropical region, promoted a new subfamily Cyrtoneurininae, and downgraded Stomoxyinae and Reinwardtiinae to tribes [13]. Fan (2008) also proposed a ten-subfamily system that was nearly the same as that of Xue and Chao (1998), except for its treatment of *Ophyra* Robineau-Desvoidy, 1830 as a valid genus which was later shown to be a junior synonym of *Hydrotaea* Robineau-Desvoidy, 1830 [14,15]. Besides those based on traditional morphological characteristics, some researchers have presented hypotheses based on molecular data. Haseyama et al. (2015) proposed a new classification of three subfamilies based on the COI, AATS, CAD, and EF1-α genes (Cyrtoneurininae, Muscinae, and Mydaeinae) [16]. Grzywacz et al. (2021) implied a classification with seven subfamilies, which were divided into two clades based on a re-interpretation of the morphology of immature stages; they raised Reinwardtiinae to a subfamily [17]. 

With developing sequencing technology, usage of gene markers has increased for muscid phylogenetic relationship reconstruction, from multi-locus [16,17,18,19] to whole genome level [17]. However, the muscid phylogenetic relationships established by mitochondrial genome data are still incomplete. Mitochondrial genomes (mtgenomes) have been shown to be effective and powerful markers for phylogenetic relationship reconstruction [20,21,22,23]. The utilities of complete mtgenomes in higher taxon-level phylogenetics have produced some remarkable achievements, e.g., recovering most deep branches of Holometabola [20,24], accurately resolving intraordinal relationships within Diptera [25], and the phylogeny and divergence time estimation of Megaloptera [21]. The Arthropoda are one of the best-studied phyla with an analysis based on mitochondrial genes [26]. Cameron et al. (2014) also demonstrated that there has been a large increase in the amount of new mtgenome data produced each year since 2005, and that the mtgenome is the most extensively studied genomic system in insects [27]. However, 88 muscid mtgenomes were accessible on the NCBI website (www.ncbi.nlm.nih.gov/nuccore, search date: 4 July 2022, keywords: Muscidae mitochondrion genome), which is not enough for understanding muscid phylogeny and evolution considering the existence of over 5000 described species in the Muscidae family.

In our study, we inferred the phylogeny of Muscidae by sequencing fifteen new mtgenomes and combined these with twenty-three available mtgenomes; all samples covered six out of seven subfamilies in Muscidae. Furthermore, we provided a mtgenomic viewpoint on the divergence time estimation based on our new phylogenetic relationships.

## 2. Materials and Methods

### 2.1. Sampling and DNA Extraction

The newly sequenced specimens were identified by Prof. Chuntian Zhang and Xin Li. The samples and taxonomic information are listed in Table 1. We also retrieved more muscid mtgenomes from the NCBI database (https://www.ncbi.nlm.nih.gov, accessed on 28 July 2021). To reduce the unbalanced sampling bias, a part of the sequences was chosen to represent species-rich and well-studied taxa. In total, there were 43 sequences sampled in our analyses, including 15 new sequenced samples, as well as 23 Muscidae sequences and five outgroup sequences retrieved from the NCBI database. The sampling covered 6 of 7 Muscidae subfamilies following the taxonomic system promoted by Carvalho et al. (2005) [13]. 

DNA was extracted from the muscle tissue of the specimen using the DNeasy Blood and Tissue Kit (Qiagen China (Shanghai) Co., Ltd.), and the DNA samples were stored at −20 °C. The DNA library procedures used a total of 0.2 g of DNA per sample as raw material. The sequencing library was created using the NEBNext^®^ UltraTM DNA Library Prep Kit for Illumina (NEB, Ipswich, MA, USA, Catalog #: E7370L) and index numbers were applied to each sample. Sonication was used to split the genomic DNA material to a size of 350 bp. Next, the DNA pieces were end polished, A-tailed, and ligated with the full-length adaptor for Illumina sequencing, which was followed by additional PCR amplification. Subsequently, the PCR results were purified using the AMPure XP technology (Beverly, MA, USA). Following this, the quality of the collection was evaluated using the Agilent 5400 system (Agilent, Santa Clara, CA, USA) and measured using QPCR (1.5 nM). The libraries were then pooled to the flow cell based on the effective concentration and target offline data volume (4–6 Gb). The cBOT was clustered and sequenced using the Illumina high-throughput sequencing platform NovaSeq 6000 with PE150 strategy in Novogene Bioinformatics Technology Co., Ltd. Beijing, China (except for OP528689, which was sequenced by the Sanger method). The raw sequence data of new mtgenomes were submitted into the SRA database with BioProject ID (PRJNA937892) and the SRA accession numbers (SRR23603891–SRR23603904) (except for OP528689). 

### 2.2. Sequences Assembly and Annotation

Data cleaning, quality control, and assembling used the “all” submodule of *MitoZ* software [29]. The genetic code was invertebrate mitochondrial code (genetic_code = 5), and the assembly was conducted with quick mode (run_mode = 2). The annotation of genes was executed in the *MITOS2* web server [42]. All protein-coding genes were checked for reading frames following the invertebrate genetic codes in *Geneious* v9 [43]. The annotations of tRNA genes were checked using the *tRNAscan-SE* Search Server (http://lowelab.ucsc.edu/tRNAscan-SE/, accessed on 31 August 2021) [44] and *ARWEN* version 1.2 (http://130.235.244.92/ARWEN/, accessed on 31 August 2021) [45]. Finally, all genes’ annotations were checked by hand following Cameron (2014) [46], who provided basic rules for annotating the mitochondrial genome.

### 2.3. Phylogeny Analyses

All dataset operations were carried out using the *Phylosuite* v1.2.2 software [47]. The alignments were executed in *MAFFT* with L-INS-I strategy [48]. In contrast with the RNA genes that were aligned through the nucleotide base, the protein-coding genes were aligned with the codon model (the MAFFT-based reading frame considering method [47]). The aligned protein-coding genes were also trimmed using *gblocks* with the codon strategy to extract conserved loci [49]. By comparing the pre-reconstructed phylogenetic results, the trimmed dataset was chosen for the following analyses. 

*PartitionFinder2* recommended the best-fit partitioning schemes (Table S1) in maximum likelihood (ML) [50]. We chose the criteria of “the corrected Akaike Information Criterion” (AICc) and the “greedy” algorithm with branch lengths estimated as “linked” to search for the optimal schemes. 

The maximum likelihood phylogenies were inferred by *IQ-TREE v1.6.8* [51]. We conducted ML analyses based on *IQ-TREE* with 5000 ultrafast bootstraps tests [52] and 1000 replications of the Shimodaira–Hasegawa-like approximate likelihood ratio test [53], and the minimum correlation coefficient was set to 0.90. The “-spp.” parameter was used to indicate that each partition could have a separate evolution rate. 

In total, 12 datasets were produced through combinations of gene markers and partition schemes (Table 2). The best result tree was used for systematic relationship discussion and divergence time estimation. Phylogenetic trees were annotated on the *iTOL* website [54].

### 2.4. Divergence Time Estimation

**Figure 1:**
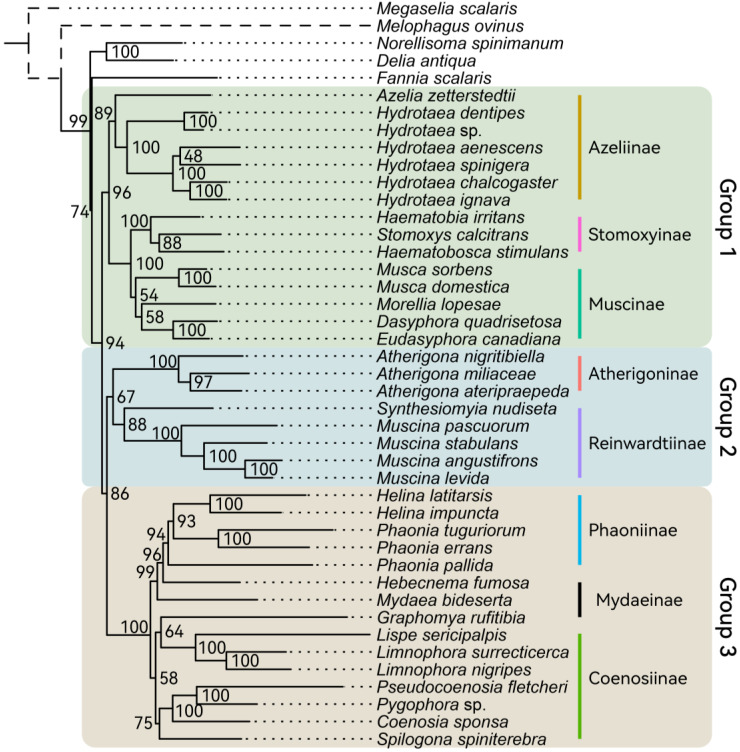
Phylogenetic analysis based on maximum likelihood method by *IQ-TREE* in *Phylosuite* software (dataset: C123TR-P2). The dashed branches were resized to better illustrate the tree.

To explore the evolutionary history, the *MCMCTree*, a software package of *PAML v4.9j*, was used to estimate the divergence time of Muscidae [55]. The relaxed-clock model (clock = 2) was used, and the divergence estimation was conducted using the quick model. First, *codeml* was used to run multiple sequence alignment (usedata = 3). Then, the approximation likelihood analyses were performed based on the previous results (usedata = 2). Four fossil calibration records were chosen from the *mindat* website (http://www.mindat.org, accessed on 31 August 2021) to estimate the divergence time within the Muscidae family: Phoridae (125–129 Ma), Muscoidea (47.8–56.0 Ma), Muscidae (46.2–50.3 Ma), and *Hydrotaea* (23.0–28.1 Ma). The phylogenetic tree of Figure 1 and the dataset “C123TR-P2PF” were used as input files to estimate the divergence time of Muscidae. By utilizing the quick model, we first set the parameter “usedata = 3”, then changed to “usedata = 2; ndata = 1; seqtype = 0; RootAge ≤ 129 Ma; nsample = 20,000; burnin = 20,000; sampfreq = 10”, while other parameters were set to default according to the user guidebook for *MCMCTree*. Two *MCMCTree* analyses were run independently to confirm that the results converged. Finally, the divergence result was parsed and shown using three R packages. The *treeio* package read the *MCMCTree* result tree [56], the *ggtree* package drew the tree [57], and the *deeptime* package illustrated the geographic time scale of the time tree [58].

## 3. Results

### 3.1. Mtgenome Assembly

There were fifteen newly sequenced mtgenomes of muscid species belonging to five subfamilies and twelve genera. We also retrieved related mtgenomes from the NCBI database, including twenty-five Muscidae species and five outgroup species from Anthomyiidae, Fanniidae, Scathophagidae, Hippoboscidae, and Phoridae. The lengths of all these mtgenomes ranged from 14,187 to 17,676 bp, and their A-T contents ranged from 77.8% to 80%. All canonical 37 genes were annotated for each mtgenome except for *Atherigona ateripraepeda* (“OP528680”, trnC missed), *Azelia zetterstedtii* (“OP528689”, trnI and trnQ missed), and *Dasyphora quadrisetosa* (“OP528694”, trnQ missed). The gene arrangements of Muscidae are similar to those found in previous dipteran mtgenome studies [59,60]. All tRNA genes were predicted and folded as cloverleaf structures.

### 3.2. Phylogenetic Effect of RNA Sequences

**Figure 2:**
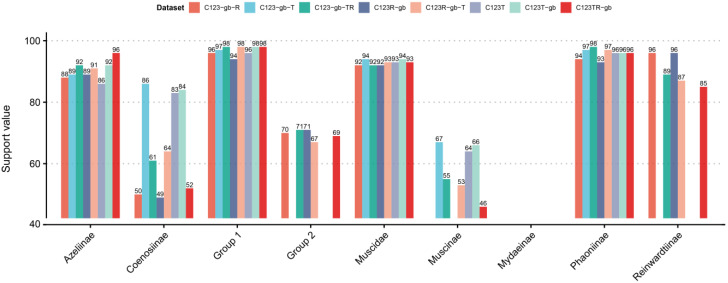
The support values of major nodes among various datasets. These nodes with same support value among datasets were hidden. The node names in x-axis were same as Figure 1. “C123”: all three loci of protein-coding gene; “T”: tRNA gene; “R”: rRNA gene. The loci followed with a “-gb” mark in the dataset name were trimmed by *gblocks*.

Studies using RNA data for phylogenetic analysis are relatively common, and some articles proved that RNA data might provide effective phylogeny signals in various groups (Diptera, Hymenoptera, Hemiptera, and Nematoda) by focusing on different systematic levels of phylogenetic relationships (from tribe to phylum) [22,61,62,63]. We found that the genetic differences in tRNA were not significant, and the trimmed RNAs were reduced by 3–28% (C123: 1–16%). We also examined the effects of using RNA data and trimming by *gblocks* in phylogenetic analyses (Figure 2). There are no significant phylogenetic differences in RNA sequences trimmed by *gblocks*. Adding both tRNA and rRNA genes rather than a single type of RNA noticeably improved the resolution of the Muscidae phylogenetic tree. 

### 3.3. Phylogenetic Analyses of Muscidae

#### 3.3.1. Phylogenetic Analyses Based on IQ-Tree

**Figure 3:**
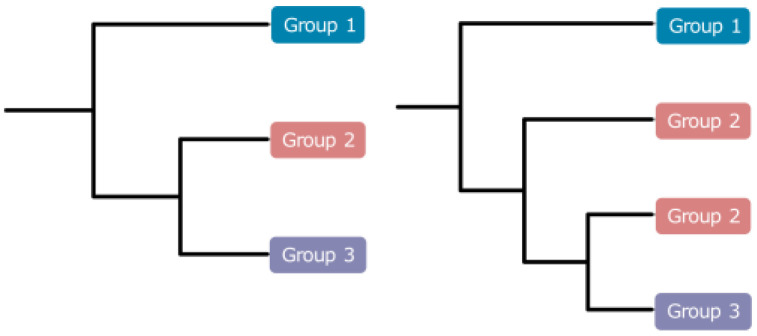
Two different tree topologies across all analyses. Group 1 represents the Azeliinae, Muscinae, and Stomoxyinae. Group 2 represents Atherigoninae and Reinwardtiinae. Group 3 represents Mydaeinae, Phaoniinae, and Coenosiinae.

To obtain the most consentaneous phylogeny, we utilized 12 datasets based on different genes or codon loci combinations (Table 2). Comparing all the results, we hypothesized that Muscidae were divided into three major groups: group one containing subfamilies Azeliinae, Muscinae and Stomoxyinae; group two consisting of subfamilies Atherigoninae and Reinwardtiinae; group three comprising subfamilies Mydaeinae, Phaoniinae, and Coenosiinae (Figure 1). Two topologies appeared across the result trees (Figure 3). The major difference was whether group two was monophyletic or not. Ten of the twelve trees built in IQ-Tree supported tree topology one, and the other two trees supported topology two (Figure 1 and Figure 3). Topology two did not cluster Atherigoninae and Reinwardtiinae as a monophyletic clade. In group one, Azeliinae were a sister group to Muscinae *sensu lato* (Muscinae and Stomoxyinae) in all IQ-Tree analyses, and these three subfamilies were recovered as monophyletic groups in most of the IQ-Tree results except for Muscinae in datasets “C123R-P2”, “C12R-P2”, and “C12TR-P2” (Table 2). In group two, most of the datasets supported the sister relationship between Atherigoninae and Reinwardtiinae, while “C123T-P1” and “C123T-P2” recovered them as paraphyletic groups. The Reinwardtiinae were separated: Atherigoninae along with the species *Synthesiomyia nudiseta* (Reinwardtiinae) and the remaining species of Reinwardtiinae. In group three, the phylogenetic relationships among three subfamilies were poorly reconstructed (Coenosiinae, Mydaeinae, and Phaoniinae). Nearly all datasets indicated Phaoniinae were monophyletic. The paraphyletic Phaoniinae and two species of Mydaeinae were mixed in two datasets (“C12T-P2” and “C12R-P2”), and one Mydaeinae species (*Graphomya rufitibia*) always nested in Coenosiinae in all datasets. When *Graphomya rufitibia* was taken out of consideration, eight datasets supported the monophyly of Coenosiinae. Mydaeinae were a non-monophyletic group in all datasets, and the two Mydaeinae species (except for *Graphomya rufitibia*) were close to each other but not as a clade in all IQ-Tree results.

#### 3.3.2. The Best Phylogenetic Tree

The best tree was ultimately determined to be the one with the highest support values. The “C123TR-P2PF” dataset with thirteen protein-coding genes, twenty-two tRNAs, and two rRNAs, and each locus of the protein-coding genes, were partitioned separately; this was used to estimate the best tree. As shown in Figure 1, Muscidae consisted of three major groups (Figure 2 and Figure 3): Azeliinae, Muscinae, and Stomoxyinae were in group one; Atherigoninae and Reinwardtiinae were in group two; and Mydaeinae, Phaoniinae, and Coenosiinae were in group three. In a few phylogenetic trees, Reinwardtiinae were assigned to group three (topology two in Figure 3). Most of the analyses supported the phylogenetic relationship of topology one: group one + (group two + group three). Therefore, we assumed that this tree topology tends to be stable.

### 3.4. Time Frame Based on Mtgenomes

**Figure 4:**
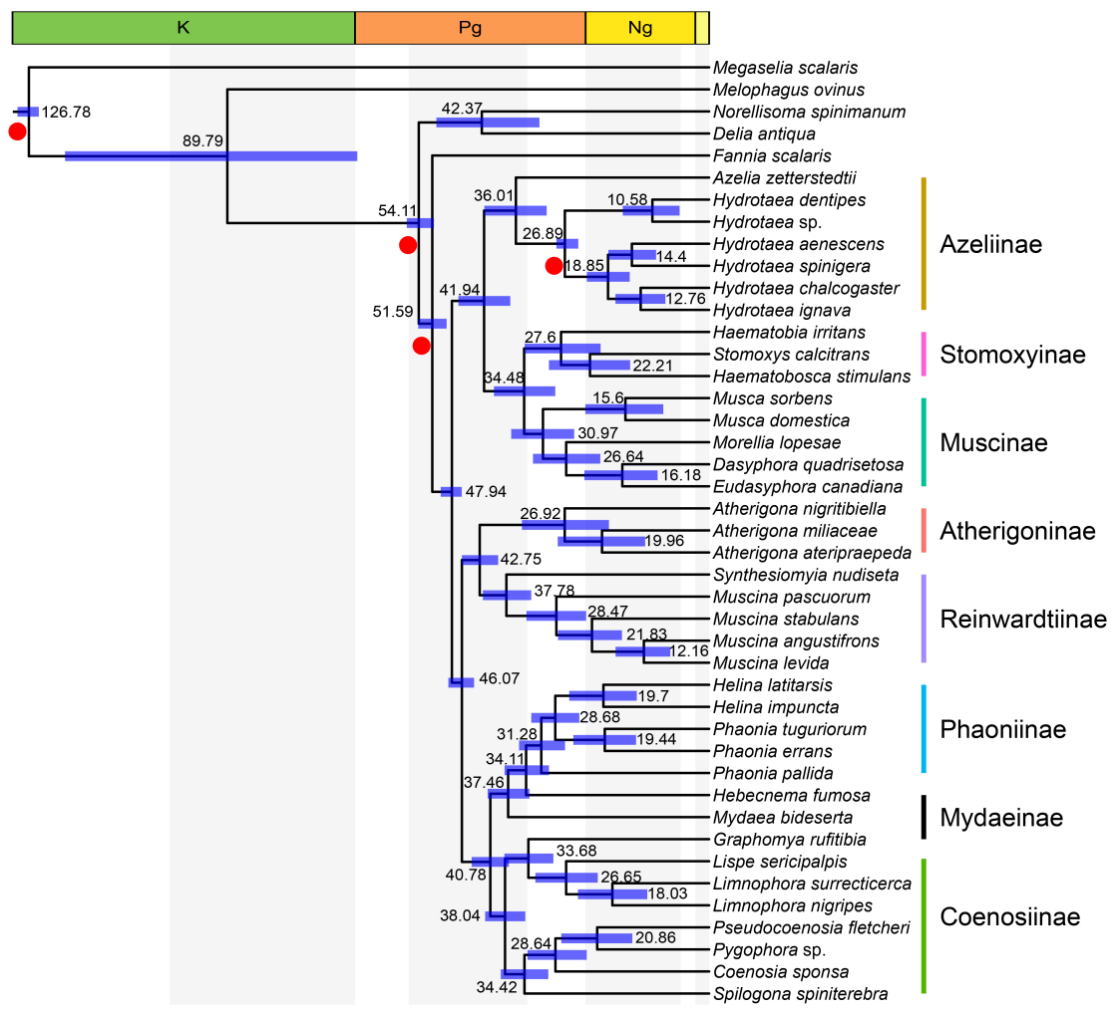
Estimation of Muscidae divergence time by *MCMCTree*. Different vertical color stripes represent subfamilies in Muscidae; the red points on nodes are time calibration records.

The result revealed that Muscidae diverged from the rest of the muscoid grade at 51.59 Ma (95% HPD: 48.93–54.22 Ma) (Figure 4), which is similar to the proposed time frame of all major Muscidae lineages [16]. First, group one (defined in Figure 1) split with groups two and three at 47.94 Ma (95%HPD: 46.10–50.03 Ma); then the split between group two and group three was dated to 46.07 Ma (95%HPD: 43.81–48.60 Ma). The Azeliinae were split from Muscinae and Stomoxyinae at 41.94 Ma (95%HPD: 37.06–46.73 Ma). The Stomoxyinae split with Muscinae at 34.48 Ma (95%HPD: 28.68–40.10 Ma). The subfamily Atherigoninae and Reinwardtiinae diverged at 42.75 Ma (95%HPD: 39.32–46.07 Ma). Coenosiinae diverged with Mydaeinae and Phaoniinae at 40.78 Ma (95%HPD: 37.33–44.26 Ma), and the split between Phaoniinae (*Mydaea* Robineau-Desvoidy, 1830 and *Hebecnema* Schnabl, 1889) and Mydaeinae occurred at 34.11 Ma (95%HPD: 29.85–38.12 Ma).

## 4. Discussion

We confirmed the early divergence of Muscidae based on mtgenomic data, and also reported the split of Muscidae into three major groups (Figure 1 and Figure 3) which were discovered in previous studies through multi-markers (mitochondrial and nuclear genes) [16,17,18], AHE, and RAD-seq datasets [17]. The morphological characteristics and molecular data provide conclusive evidence for the monophyly of Muscidae [11,16,17,18,19,64,65,66]. However, the monophylies of some subfamilies (i.e., Azeliinae *sensu lato* (including Reinwardtiinae), Muscinae *sensu lato* (including Stomoxyinae), Mydaeinae, and Phaoniinae) have not been fully supported [16,17,18,65,66,67]. 

The divisions of subfamilies Azeliinae *sensu lato* (including Azeliinae and Reinwardtiinae) and Muscinae *sensu lato* (including Muscinae and Stomoxyinae) in Muscidae were inconsistent in the past. Couri and Carvalho (2003) revealed the cladistic relationship of seven subfamilies and rebuilt Muscinae *sensu lato* as a sister group to the rest of the subfamilies (Muscinae + (Azeliinae + (Phaoniinae + (Reinwardtiinae + (Dichaetomyiinae + (Mydaeinae + Coenosiinae)))))) [64]. Schuehli et al. (2007) pointed out that subfamily Azeliinae *sensu lato* were paraphyletic and mixed with the polyphyletic Muscinae *sensu lato* in their strict consensus tree of combined data for four gene markers [19]. However, Grzywacz et al. (2017) considered that their gene trees were anomalous and that the position of *Hydrotaea* and *Ophyra* within Azeliinae could not be supported [15]. Kutty et al. (2008, 2010) offered phylogenetic analyses with eight gene markers; they obtained a split between genus *Muscina* Robineau-Desvoidy, 1830 (Reinwardtiinae) and the rest of Azeliinae *sensu lato*, and their results failed to support a sister-group relationship between Muscinae and Stomoxyinae [65,66]. With complete tribal sampling, Kutty et al. (2014) also confirmed the clear split between Azeliinae and Reinwardtiinae as well as the non-sister-group relationship between Muscinae and Stomoxyinae [18]. Haseyama et al. (2015) proposed a new classification where Muscidae consisted of three subfamilies (Muscinae + (Cyrtoneurininae + Mydaeinae)), which represented all tribes of Muscidae and all biogeographic regions [16]. The phylogeny showed a significant cladistic distance between Azeliini and Reinwardtiini and rejected the monophyly of Azeliinae. They also provided a time frame based on four gene markers: the housefly subfamilies originated during Paleocene to Eocene. Ding et al. (2015) indicated that Muscidae split from Fanniidae at 37.65 Ma (late Eocene) based on three muscid mtgenomes [22]. Ren et al. (2019) inferred phylogenetic relationships within Muscidae based on fifteen mtgenomes of four subfamilies and recovered Mydaeinae as a basal clade: Mydaeinae + (Reinwardtiinae + (Muscinae *sensu lato* + Azeliinae)) [23]. Kutty et al. (2019) corroborated the monophyly of Muscinae *sensu lato* and Coenosiinae and revealed a close relationship between Mydaeinae and Phaoniinae in their Calyptratae phylogenetic research [67]. However, their result was not as comprehensive as their previous two studies [16,18]. Grzywacz et al. (2021) rearranged the classification of Muscidae based on multiple molecular markers and presented a topology that partially contradicts the traditional classification based on adult morphology; however, it is to some extent consistent with larval morphology [17]. The Reinwardtiini were resurrected as subfamily Reinwardtiinae; four genera previously settled in Eginiini were transferred to Reinwardtiinae; and Stomoxyini were still placed in Muscinae. The split of Muscidae into three major groups (defined in Figure 1 and Figure 3) was also documented among their various datasets [17].

The monophyly of Azeliinae *sensu lato* depends on whether Reinwardtiinae are included. Azeliinae were placed under the Muscinae *sensu lato* or Muscinae [68,69,70], and raised as a subfamily based on adult morphology by Carvalho (1989) [11]. The monophyly of Azeliinae *sensu lato* was rejected by the cladogram of 54 morphological traits; the significant differences between Azeliinae and Reinwardtiinae in musculature of the male terminalia [64,71]; and the non-monophyletic position in molecular phylogenetic studies [16,17,18]. The subfamily status of Azeliinae and Reinwardtiinae was supported [3,12,64], while the Azeliinae was also recovered as *Azelia* + (Azeliinae + Muscinae/Muscinae *sensu lato*) [15,16,17]. In addition to the relationship between Azeliinae and Reinwardtiinae, their research also suggested the basal position of Azeliinae within Muscidae. Since *Azelia* Robineau-Desvoidy, 1830 was considered within Azeliini (as Hydrotaeini) by Hennig (1965) [69], *Azelia* was placed close to *Hydrotaea* and *Thricops* Rondani, 1856. Haseyama et al. (2015) recovered *Azelia* within *Hydrotaea* and as a sister to *Ophyra* (revised as an invalid genus later and placed in *Hydrotaea* [15]) [16]. Savage and Wheeler (2004) showed that *Azelia* was close to *Thricops* instead of *Hydrotaea* in their phylogenetic tree inferred by morphological traits [72]. The close relationship of these genera was questioned by Grzywacz et al. (2017) and the *Azelia* was recovered as sister group to the remaining Azeliinae + Muscinae *sensu lato* [15,17]. Different from previous studies, the species of *Azelia* were rebuilt within Azeliinae and the monophyly of Azeliinae was recovered in the present study [15,16,17]. It should be noted that the genera *Thricops* and *Huckettomyia* Pont *et* Shinonaga, 1970 of Azeliinae were not included in our analyses. These two genera were recovered as a sister group to Muscinae/Muscinae *sensu lato* [15,16,17], but were recovered within Azeliinae in studies of Kutty et al. [18,65,66]. However, it is challenging to identify whether the sampling of *Thricops* and *Huckettomyia* has a significant impact on the reconstruction of Azeliinae monophyly or not.

Whether at the gene fragment level (nuclear and mitochondrial gene fragments [16,18,19,65,66]) or the genome level (anchored hybrid enrichment and restriction site-associated DNA sequencing at the genomic scale [17]), Azeliinae *sensu lato* were rebuilt as a non-monophyletic lineage. The majority of studies did not support the monophyly of Azeliinaeae *sensu lato* [16,17,18,65,66]. Only two species of Reinwardtiinae were grouped by Kutty et al. [65,66]. The poorly sampled Reinwardtiinae were clustered with Coenosiinae + (Mydaeinae, Phaoniinae) [65,66]; alternatively, Reinwardtiinae were polyphyletic and were grouped with Phaoniinae, Cyrtoneurininae, and even Mydaeinae [16,17,18]. Following the clues mentioned above, most studies supported the divergence between Azeliinae and Reinwardtiinae, and several studies claimed the subfamily status of Azeliinae (Azeliini) and Reinwardtiinae (Reinwardtiini). However, few molecular studies supported the monophyly of these two subfamilies, and no result supported their sister-group relationship. Unlike previous hypotheses, based on the sampling in the present study, our results supported the monophyly of Azeliinae and Reinwardtiinae, independently. They might be raised as subfamilies; further studies that concentrate on morphology, reproductive strategy, and behavior are needed to provide more pieces of evidence. 

The monophyly of Muscinae was supported by many studies [16,18,19,64,67]. Previously, Stomoxyinae were classified in Muscinae *sensu lato* [11,17,18,64,65,67,73], and their monophyly was well supported. However, the position of Stomoxyinae in Muscinae was inconsistent. In some studies, Stomoxyinae were close to the base of Muscinae *sensu lato* [14,64,66], while more studies have favored Stomoxyinae to be nested within the Muscinae *sensu lato* in a distal position [16,17,18,65]. In opposition to some previous studies, our phylogenetic results support Stomoxyinae as a sister group to Muscinae. In terms of morphological aspects, Stomoxyinae were distinguished from Muscinae in the number of spermatheca (two versus three), type of mouthparts (piercing sucking versus sponging), and shape of arista (pectinate versus plumose) [14]. The haustellum of Stomoxyinae is strongly sclerotized [12] and Stomoxyinae are blood-feeding insects [7,74]. We favor the family rank of Stomoxyinae based on these morphological characteristics and the sister-group relationship between Muscinae and Stomoxyinae. 

There is no doubt that Atherigoninae were monophyletic [18,73]. The only question is their systematic position. They were placed close to the base of Muscidae [11] and are closely related to Reinwardtiinae in taxonomic revisions [14]. The sister-group relationship to the Reinwardtiinae was also favored by the anchored hybrid enrichment dataset [17], while the Atherigoninae were clustered with Cyrtoneurininae by fewer than eight molecular markers [16,18]. 

Coenosiinae have usually been considered as a monophyletic group [17,18,64,65,66,67]. Phaoniinae, Mydaeinae, and Coenosiinae were often clustered in one clade based on morphological characteristics [11,18,65,66,67]. However, Phaoniinae were grouped with Azeliinae and Reinwardtiinae on the basis of the cilia absent on sternite one [64]. The relationship between Phaoniinae and Mydaeinae (*Graphomya*, *Hebecnema*, and *Mydaea*) as well as the monophylies of Phaoniinae and Mydaeinae were poorly supported in recent studies [16,17,18,65,66,67]. Except for clustering with Phaoniinae, Mydaeinae were also rebuilt close to Coenosiinae or Reinwardtiinae. The non-monophyletic group Mydaeinae (consisting of genera *Gymnodia* Robineau-Desvoidy, 1830 and *Graphomya*) were clustered alongside Coenosiinae under the analyses of anchored hybrid enrichment (AHE) data [17]. The genus *Graphomya* was close to Limnophorini (Coenosiinae), and the genera *Hebecnema* and *Mydaea* were grouped with Phaoniinae by four genes [16]. The Mydaeinae represented by *Graphomya rufitibia* alone were grouped with Reinwardtiinae or Reinwardtiinae + (Muscinae + Azeliinae) by mtgenomes [23,41,75]. Those analyses lacked data from Phaoniinae and Coenosiinae, which may cause the bias in phylogenetic reconstruction. The *Graphomya* of Mydaeinae was once assigned to Coenosiinae because the dark brown spots on their abdomen were similar to those of *Limnophora* Robineau-Desvoidy, 1830 and *Spilogona* Schnabl, 1911 [12]. *Graphomya* was also clustered with Coenosiinae in our phylogenetic tree. The mixed relationship among Coenosiinae, Phaoniinae, and Mydaeinae was similar to our findings; however, Coenosiinae and Phaoniinae were monophyletic rather than polyphyletic based on our study. To summarize, although we cannot directly determine whether or not *Graphomya*, *Mydaea,* and *Hebecnema* of Mydaeinae should be reorganized into Coenosiinae and Phaoniinae, our study supports that Mydaeinae is closer to Phaoniinae than Coenosiinae [16,18,65,66].

The facts that the genera *Helina* Robineau-Desvoidy, 1830 and *Phaonia* Robineau-Desvoidy, 1830 were grouped in our phylogenetic tree and that Kutty et al. (2014) observed a similar link between the two genera [18], suggests that *Helina* should be synonymized with *Phaonia*. This theory, however, was still being debated. *Phaonia* and *Helina* were paraphyletic groups and both need significant revision through redefinition or combination [5,76]. However, Kutty et al. (2014) and Ma et al. (2002) suggested separating these two groups based on the presence of a strong posterodorsal seta on the hind tibia in *Phaonia* which constituted an undeniable symplesiomorphy [18,77]. 

The divergence time of Muscidae was estimated based on the best tree (Figure 1) under the quick mode (see details in methods). Our results showed that the divergence between Muscidae and ((Scathophagidae + Anthomyiidae) + Fanniidae) occurred in the early Eocene (51.59 Ma, 95% HPD: 48.93–54.22 Ma), which was close to the estimation of previous findings (from 34.9 Ma to 51.5 Ma) [16,22,35,78,79]. Azeliinae diverged with Muscinae and Stomoxyinae approximately at 41.94 Ma (95%HPD: 37.06–46.73 Ma), which was also close to the previous estimation (39.41 Ma, 95%HPD: 32.5–44.54 Ma) [35]. The time span of Stomoxyinae’s split with Muscinae at 34.48 Ma (95%HPD: 28.68–40.10 Ma) overlaps with the interval of 22–40 Ma estimated by Dsouli et al. (2011) [79] and 19–32 Ma estimated by Haseyama et al. (2015) [16]. It is worth mentioning that the divergence time of Muscidae is located in the early Eocene climatic optimum period (EECO, ca. 51–53 Ma, a period of peak global warmth) [80], and most of the subfamilies diverged around the middle Eocene climatic optimum period (MECO, 40–41.5 Ma, another period of peak global warmth in the secular Cenozoic cooling trend) [81,82]. Löwenberg-Neto et al. (2020) suggested the Neotropical region as the ancestral area of Muscidae, which then spread to the Palearctic region [83]. Until now, there is no evidence to support the claims that the EECO or MECO events contributed to the early diversification of muscids in the Neotropical region. 

Our study provides a mtgenomic view of Muscidae evolutionary history. Our estimation of Muscidae has relatively narrow confidence intervals. At present, few studies are focusing on Muscidae divergence time estimation [16,79] and no study uses a mtgenome strategy to estimate the time frame of Muscidae subfamilies. Here, we reconstructed the phylogenetic relationship among Muscidae subfamilies based on a relatively improved sampling of the mtgenome at the subfamily level. In general, our phylogenetic results indicated that the relationship among subfamilies was similar to previous studies. Our mtgenome strategy offers strong support in the phylogenetic relationships of the Muscidae subfamily. Furthermore, our study clarifies the monophyly of subfamilies among Muscidae. 

## Figures and Tables

**Table 1:** Information of sampled sequences and their systematic ranks were used in this study. The “-” means that we cannot find its reference in both NCBI website and internet.

Subfamily	Tribe	Organism	Accession Number	Reference
Outgroup		*Delia antiqua* (Meigen, 1826)	NC_028226	[28]
		*Fannia scalaris* (Fabricius, 1794)	NC_053661	[29]
		*Megaselia scalaris* (Loew, 1866)	KF974742	[30]
		*Melophagus ovinus* (Linnaeus, 1758)	MH024396	[31]
		*Norellisoma spinimanum* (Fallén, 1819)	NC_050316	-
Atherigoninae	Atherigoniini	*Atherigona ateripraepeda* He, Huang *et* Fang, 2007	OP528680	This study
		*Atherigona miliaceae* Malloch, 1925	OP528691	This study
		*Atherigona nigritibiella* Fan *et* Liu, 1982	OP528682	This study
Azeliinae	Azeliini	*Azelia zetterstedtii* Rondani, 1866	OP528689	This study
		*Hydrotaea aenescens* (Wiedemann, 1830)	NC_042952	[23]
		*Hydrotaea chalcogaster* (Wiedemann, 1824)	NC_041089	[32]
		*Hydrotaea dentipes* (Fabricius, 1805)	NC_047403	[33]
		*Hydrotaea ignava* (Harris, 1780)	NC_037195	[34]
		*Hydrotaea* sp. Robineau-Desvoidy, 1830	KT272841	[35]
		*Hydrotaea spinigera* Hennig, 1962	NC_042951	[23]
	Reinwardtiini	*Muscina angustifrons* (Loew, 1858)	NC_034805	[36]
		*Muscina levida* (Harris, 1780)	NC_029487	[35]
		*Muscina pascuorum* (Meigen, 1826)	NC_053670	[37]
		*Muscina stabulans* (Fallén, 1817)	NC_026292	[38]
		*Synthesiomyia nudiseta* (van der Wulp, 1883)	NC_042953	[23]
Coenosiinae	Coenosiini	*Coenosia sponsa* Xue *et* Tong, 2004	OP528683	This study
		*Pseudocoenosia fletcheri* (Malloch, 1919)	OP528679	This study
		*Pygophora* sp. Schiner, 1868	OP528684	This study
	Limnophorini	*Limnophora nigripes* (Robineau Desvoidy, 1830)	OP528687	This study
		*Limnophora surrecticerca* Xue *et* Zhang, 1998	OP528690	This study
		*Spilogona spiniterebra* (Stein, 1907)	OP528685	This study
	Lispini	*Lispe sericipalpis* Stein, 1904	OP528692	This study
Muscinae	Muscini	*Dasyphora quadrisetosa* Zimin, 1951	OP528694	This study
		*Eudasyphora canadiana* Cuny, 1980	KT272852	[35]
		*Morellia lopesae* Pamplona, 1986	KT272863	[35]
		*Musca domestica* Linnaeus, 1758	NC_024855	[39]
		*Musca sorbens* Wiedemann, 1830	NC_037910	[40]
	Stomoxyini	*Haematobia irritans* (Linnaeus, 1758)	NC_007102	-
		*Haematobosca stimulans* (Meigen, 1824)	MT410787	-
		*Stomoxys calcitrans* (Linnaeus, 1758)	OP528693	This study
Mydaeinae	Mydaeini	*Graphomya rufitibia* Stein, 1918	NC_038210	[41]
		*Hebecnema fumosa* (Meigen, 1826)	OP528688	This study
		*Mydaea bideserta* Xue *et* Wang, 1992	OP528681	This study
Phaoniinae	Phaoniini	*Helina impuncta* (Fallén, 1823)	MT410825	-
		*Helina latitarsis* Ringdahl, 1924	MT410783	-
		*Phaonia errans* (Meigen, 1826)	MT920423	-
		*Phaonia pallida* (Fabricius, 1787)	MT584137	-
		*Phaonia tuguriorum* (Scopoli, 1763)	MT410813	-

**Table 2:** Datasets used for Muscidae tree reconstruction. The asterisk indicates the best dataset for phylogenetic analyses in our study.

	Dataset Name	Protein-Coding Genes	Partition Scheme	tRNAs	rRNAs
1	C123-P1	all three codons	by gene		
2	C123-P2	all three codons	by locus		
3	C123T-P1	all three codons	by gene	22 tRNAs	
4	C123T-P2	all three codons	by locus	22 tRNAs	
5	C123R-P1	all three codons	by gene		2 rRNAs
6	C123R-P2	all three codons	by locus		2 rRNAs
7	C123TR-P1	all three codons	by gene	22 tRNAs	2 rRNAs
8	C123TR-P2 *	all three codons	by locus	22 tRNAs	2 rRNAs
9	C12-P2	remove 3rd codon	by locus		
10	C12T-P2	remove 3rd codon	by locus	22 tRNAs	
11	C12R-P2	remove 3rd codon	by locus		2 rRNAs
12	C12TR-P2	remove 3rd locus	by locus	22 tRNAs	2 rRNAs

## Data Availability

The fifteen newly sequenced mtgenomes were submitted to the GenBank database under the accession numbers OP528679–OP528694 (except for OP528686). The raw sequence data of new mtgenomes were submitted to the SRA database with SRA accession numbers SRR23603891–SRR23603904 (except for OP528689 that sequenced by Sanger method).

## Supplementary Materials

The following supporting information can be downloaded at: https://www.mdpi.com/article/10.3390/insects14030286/s1, Table S1: The genes combination and their partition and models used for phylogenetic analyses.
