# Supplementary material for: Phylogeny and Evolutionary Timescale of Muscidae (Diptera: Calyptratae) Inferred from Mitochondrial Genomes
Source: Insects. 2023 Mar 15;14(3):286. doi: 10.3390/insects14030286 (PMC10059281; doi:10.3390/insects14030286)
Supplement: Supplementary file 1 [file insects-14-00286-s001.zip › insects-2181505-supplementary.pdf]

Supplementary

Table S1. The gene combination, their partition, and models used for phylogenetic analyses. C123: the 13 protein coding genes (PCGs) with all three loci; C12: the protein coding genes with the first and second locus; T: the 22 tRNAs; R: the 2 rRNAs; P1: indicated that the 13 PCGs were partitioned by gene; P2: indicated that the 13 PCGs were partitioned by locus; partitions and models were inferred by PartitionFinder2 [50].

Partitions	IQ-TREE models	MrBayes models
C123-P1		
Subset1 = 3046−3831, 1−675	GTR + I+G:Subset1,	lset applyto = (1) nst = 6 rates = invgamma;
Subset2 = 10618−11139, 676−831	GTR + I+G:Subset2,	lset applyto = (2) nst = 6 rates = invgamma;
Subset3 = 832−2361	GTR + I+G:Subset3,	lset applyto = (3) nst = 6 rates = invgamma;
Subset4 = 2362−3045	GTR + I+G:Subset4,	lset applyto = (4) nst = 6 rates = invgamma;
Subset5 = 3832−4965, 6922−7272	GTR + I+G:Subset5,	lset applyto = (5) nst = 6 rates = invgamma;
Subset6 = 4966−5907	GTR + I+G:Subset6,	lset applyto = (6) nst = 6 rates = invgamma;
Subset7 = 5908−6921	TIM + I+G:Subset7,	lset applyto = (7) nst = 6 rates = invgamma;
Subset8 = 7273−7560	K81UF + G:Subset8,	lset applyto = (8) nst = 6 rates = gamma;
Subset9 = 7561−8898	GTR + I+G:Subset9,	lset applyto = (9) nst = 6 rates = invgamma;
Subset10 = 8899−10617	GTR + I+G:Subset10;	lset applyto = (10) nst = 6 rates = invgamma;
C123-P2		
Subset1 = 1−675\3, 6922−7272\3	GTR + I+G:Subset1,	lset applyto = (1) nst = 6 rates = invgamma;
Subset2 = 2363−3045\3, 2−675\3	TVM + I+G:Subset2,	lset applyto = (2) nst = 6 rates = invgamma;
Subset3 = 10620−11139\3, 3−675\3, 6924−7272\3	TRN + I+G:Subset3,	lset applyto = (3) nst = 6 rates = invgamma;
Subset4 = 676−831\3	TIM + I+G:Subset4,	lset applyto = (4) nst = 6 rates = invgamma;
Subset5 = 677−831\3	GTR + G:Subset5,	lset applyto = (5) nst = 6 rates = gamma;
Subset6 = 678−831\3	GTR + G:Subset6,	lset applyto = (6) nst = 6 rates = gamma;
Subset7 = 832−2361\3	GTR + I+G:Subset7,	lset applyto = (7) nst = 6 rates = invgamma;
Subset8 = 833−2361\3	TVM + I+G:Subset8,	lset applyto = (8) nst = 6 rates = invgamma;
Subset9 = 834−2361\3, 5910−6921\3	TIM + G:Subset9,	lset applyto = (9) nst = 6 rates = gamma;
Subset10 = 2362−3045\3	GTR + I+G:Subset10,	lset applyto = (10) nst = 6 rates = invgamma;
Subset11 = 2364−3045\3	HKY + I+G:Subset11,	lset applyto = (11) nst = 2 rates = invgamma;
Subset12 = 3832−4965\3, 3046−3831\3	GTR + I+G:Subset12,	lset applyto = (12) nst = 6 rates = invgamma;
Subset13 = 3047−3831\3, 3833−4965\3	GTR + I+G:Subset13,	lset applyto = (13) nst = 6 rates = invgamma;
Subset14 = 3048−3831\3, 3834−4965\3	TIM + I+G:Subset14,	lset applyto = (14) nst = 6 rates = invgamma;
Subset15 = 4966−5907\3	GTR + I+G:Subset15,	lset applyto = (15) nst = 6 rates = invgamma;
Subset16 = 4967−5907\3	K81UF + I+G:Subset16,	lset applyto = (16) nst = 6 rates = invgamma;
Subset17 = 7275−7560\3, 7563−8898\3, 4968−5907\3, 8901−10617\3	GTR + I+G:Subset17,	lset applyto = (17) nst = 6 rates = invgamma;
Subset18 = 5908−6921\3	GTR + I+G:Subset18,	lset applyto = (18) nst = 6 rates = invgamma;
Subset19 = 5909−6921\3	GTR + I+G:Subset19,	lset applyto = (19) nst = 6 rates = invgamma;
Subset20 = 6923−7272\3	TVM + I+G:Subset20,	lset applyto = (20) nst = 6 rates = invgamma;
Subset21 = 7273−7560\3	TVM + G:Subset21,	lset applyto = (21) nst = 6 rates = gamma;
Subset22 = 7274−7560\3	K81UF + G:Subset22,	lset applyto = (22) nst = 6 rates = gamma;
Subset23 = 7561−8898\3	TVM + I+G:Subset23,	lset applyto = (23) nst = 6 rates = invgamma;
Subset24 = 8900−10617\3, 7562−8898\3	GTR + I+G:Subset24,	lset applyto = (24) nst = 6 rates = invgamma;
Subset25 = 8899−10617\3	GTR + I+G:Subset25,	lset applyto = (25) nst = 6 rates = invgamma;
Subset26 = 10618−11139\3	GTR + I+G:Subset26,	lset applyto = (26) nst = 6 rates = invgamma;
Subset27 = 10619−11139\3	GTR + I+G:Subset27;	lset applyto = (27) nst = 6 rates = invgamma;
C123R-P1		
Subset1 = 1−675, 3046−3831	GTR + I+G:Subset1,	lset applyto = (1) nst = 6 rates = invgamma;
Subset2 = 10618−11139, 676−831	GTR + I+G:Subset2,	lset applyto = (2) nst = 6 rates = invgamma;
Subset3 = 832−2361	GTR + I+G:Subset3,	lset applyto = (3) nst = 6 rates = invgamma;
Subset4 = 2362−3045	GTR + I+G:Subset4,	lset applyto = (4) nst = 6 rates = invgamma;
Subset5 = 3832−4965	GTR + I+G:Subset5,	lset applyto = (5) nst = 6 rates = invgamma;
Subset6 = 4966−5907	GTR + I+G:Subset6,	lset applyto = (6) nst = 6 rates = invgamma;

Subset7 = 5908–6921	TIM + I+G:Subset7,	lset applyto = (7) nst = 6 rates = invgamma;
Subset8 = 6922–7272	TIM + I+G:Subset8,	lset applyto = (8) nst = 6 rates = invgamma;
Subset9 = 7273–7560	K81UF + G:Subset9,	lset applyto = (9) nst = 6 rates = gamma;
Subset10 = 7561–8898	GTR + I+G:Subset10,	lset applyto = (10) nst = 6 rates = invgamma;
Subset11 = 8899–10617	GTR + I+G:Subset11,	lset applyto = (11) nst = 6 rates = invgamma;
Subset12 = 11140–12359	GTR + I+G:Subset12,	lset applyto = (12) nst = 6 rates = invgamma;
Subset13 = 12360–13116	GTR + G:Subset13;	lset applyto = (13) nst = 6 rates = gamma;
C123R-P2		
Subset1 = 1–675\3, 6922–7272\3	GTR + I+G:Subset1,	lset applyto = (1) nst = 6 rates = invgamma;
Subset2 = 3833–4965\3, 2–675\3, 2363–3045\3, 3047–3831\3	TVM + I+G:Subset2,	lset applyto = (2) nst = 6 rates = invgamma;
Subset3 = 10620–11139\3, 3–675\3	HKY + I+G:Subset3,	lset applyto = (3) nst = 2 rates = invgamma;
Subset4 = 676–831\3	TIM + I+G:Subset4,	lset applyto = (4) nst = 6 rates = invgamma;
Subset5 = 677–831\3	GTR + G:Subset5,	lset applyto = (5) nst = 6 rates = gamma;
Subset6 = 6924–7272\3, 678–831\3	GTR + I+G:Subset6,	lset applyto = (6) nst = 6 rates = invgamma;
Subset7 = 832–2361\3	GTR + I+G:Subset7,	lset applyto = (7) nst = 6 rates = invgamma;
Subset8 = 833–2361\3	TVM + I+G:Subset8,	lset applyto = (8) nst = 6 rates = invgamma;
Subset9 = 5910–6921\3, 834–2361\3	TIM + G:Subset9,	lset applyto = (9) nst = 6 rates = gamma;
Subset10 = 2362–3045\3	GTR + I+G:Subset10,	lset applyto = (10) nst = 6 rates = invgamma;
Subset11 = 2364–3045\3	TRN + G:Subset11,	lset applyto = (11) nst = 6 rates = gamma;
Subset12 = 3832–4965\3, 3046–3831\3	GTR + I+G:Subset12,	lset applyto = (12) nst = 6 rates = invgamma;
Subset13 = 3048–3831\3, 3834–4965\3	TIM + I+G:Subset13,	lset applyto = (13) nst = 6 rates = invgamma;
Subset14 = 4966–5907\3	GTR + I+G:Subset14,	lset applyto = (14) nst = 6 rates = invgamma;
Subset15 = 4967–5907\3	K81UF + I+G:Subset15,	lset applyto = (15) nst = 6 rates = invgamma;
Subset16 = 4968–5907\3	TRN + G:Subset16,	lset applyto = (16) nst = 6 rates = gamma;
Subset17 = 5908–6921\3	GTR + I+G:Subset17,	lset applyto = (17) nst = 6 rates = invgamma;
Subset18 = 5909–6921\3	GTR + I+G:Subset18,	lset applyto = (18) nst = 6 rates = invgamma;
Subset19 = 6923–7272\3	TVM + I+G:Subset19,	lset applyto = (19) nst = 6 rates = invgamma;
Subset20 = 7273–7560\3	TVM + G:Subset20,	lset applyto = (20) nst = 6 rates = gamma;
Subset21 = 7274–7560\3	K81UF + G:Subset21,	lset applyto = (21) nst = 6 rates = gamma;
Subset22 = 7563–8898\3, 7275–7560\3	K81UF + I+G:Subset22,	lset applyto = (22) nst = 6 rates = invgamma;
Subset23 = 7561–8898\3	TVM + I+G:Subset23,	lset applyto = (23) nst = 6 rates = invgamma;
Subset24 = 7562–8898\3	GTR + I+G:Subset24,	lset applyto = (24) nst = 6 rates = invgamma;
Subset25 = 8899–10617\3	GTR + I+G:Subset25,	lset applyto = (25) nst = 6 rates = invgamma;
Subset26 = 8900–10617\3	GTR + I+G:Subset26,	lset applyto = (26) nst = 6 rates = invgamma;
Subset27 = 8901–10617\3	K81UF + G:Subset27,	lset applyto = (27) nst = 6 rates = gamma;
Subset28 = 10618–11139\3	GTR + I+G:Subset28,	lset applyto = (28) nst = 6 rates = invgamma;
Subset29 = 10619–11139\3	TVM + I+G:Subset29,	lset applyto = (29) nst = 6 rates = invgamma;
Subset30 = 11140–12359	GTR + I+G:Subset30,	lset applyto = (30) nst = 6 rates = invgamma;
Subset31 = 12360–13116	GTR + G:Subset31;	lset applyto = (31) nst = 6 rates = gamma;
C123T-P1		
Subset1 = 3046–3831, 1–675	GTR + I+G:Subset1,	lset applyto = (1) nst = 6 rates = invgamma;
Subset2 = 676–831, 10618–11139	GTR + I+G:Subset2,	lset applyto = (2) nst = 6 rates = invgamma;
Subset3 = 832–2361	GTR + I+G:Subset3,	lset applyto = (3) nst = 6 rates = invgamma;
Subset4 = 2362–3045	GTR + I+G:Subset4,	lset applyto = (4) nst = 6 rates = invgamma;
Subset5 = 3832–4965	GTR + I+G:Subset5,	lset applyto = (5) nst = 6 rates = invgamma;
Subset6 = 11513–11576, 4966–5907	GTR + I+G:Subset6,	lset applyto = (6) nst = 6 rates = invgamma;
Subset7 = 5908–6921	GTR + I+G:Subset7,	lset applyto = (7) nst = 6 rates = invgamma;
Subset8 = 6922–7272	TIM + I+G:Subset8,	lset applyto = (8) nst = 6 rates = invgamma;
Subset9 = 7273–7560, 11205–11264	K81UF + G:Subset9,	lset applyto = (9) nst = 6 rates = gamma;
Subset10 = 7561–8898	GTR + I+G:Subset10,	lset applyto = (10) nst = 6 rates = invgamma;
Subset11 = 8899–10617	GTR + I+G:Subset11,	lset applyto = (11) nst = 6 rates = invgamma;
Subset12 = 11140–11204, 11454–11512, 11760–11825	TVM + I+G:Subset12,	lset applyto = (12) nst = 6 rates = invgamma;
Subset13 = 12288–12351, 11265–11327	GTR + I+G:Subset13,	lset applyto = (13) nst = 6 rates = invgamma;
Subset14 = 11895–11958, 11328–11388	TVM + I+G:Subset14,	lset applyto = (14) nst = 6 rates = invgamma;
Subset15 = 11389–11453, 12024–12092	HKY + G:Subset15,	lset applyto = (15) nst = 2 rates = gamma;

Subset16 = 11577–11630, 12487–12551	TVM + G:Subset16,	lset applyto = (16) nst = 6 rates = gamma;
Subset17 = 11631–11701, 11826–11894	TVM + I+G:Subset17,	lset applyto = (17) nst = 6 rates = invgamma;
Subset18 = 11702–11759	HKY + G:Subset18,	lset applyto = (18) nst = 2 rates = gamma;
Subset19 = 12352–12420, 11959–12023	TIM + G:Subset19,	lset applyto = (19) nst = 6 rates = gamma;
Subset20 = 12093–12154, 12223–12287	GTR + I+G:Subset20,	lset applyto = (20) nst = 6 rates = invgamma;
Subset21 = 12155–12222, 12421–12486	TVM + G:Subset21;	lset applyto = (21) nst = 6 rates = gamma;
C123T-P2		
Subset1 = 1–675\3, 6922–7272\3	GTR + I+G:Subset1,	lset applyto = (1) nst = 6 rates = invgamma;
Subset2 = 2363–3045\3, 2–675\3	TVM + I+G:Subset2,	lset applyto = (2) nst = 6 rates = invgamma;
Subset3 = 2364–3045\3, 3–675\3, 6924–7272\3	TVM + I+G:Subset3,	lset applyto = (3) nst = 6 rates = invgamma;
Subset4 = 676–831\3	TIM + I+G:Subset4,	lset applyto = (4) nst = 6 rates = invgamma;
Subset5 = 677–831\3	GTR + G:Subset5,	lset applyto = (5) nst = 6 rates = gamma;
Subset6 = 678–831\3	GTR + G:Subset6,	lset applyto = (6) nst = 6 rates = gamma;
Subset7 = 832–2361\3	GTR + I+G:Subset7,	lset applyto = (7) nst = 6 rates = invgamma;
Subset8 = 833–2361\3	TVM + I+G:Subset8,	lset applyto = (8) nst = 6 rates = invgamma;
Subset9 = 834–2361\3, 5910–6921\3	TIM + G:Subset9,	lset applyto = (9) nst = 6 rates = gamma;
Subset10 = 2362–3045\3, 11577–11630	GTR + I+G:Subset10,	lset applyto = (10) nst = 6 rates = invgamma;
Subset11 = 3832–4965\3, 3046–3831\3	GTR + I+G:Subset11,	lset applyto = (11) nst = 6 rates = invgamma;
Subset12 = 3833–4965\3, 3047–3831\3	GTR + I+G:Subset12,	lset applyto = (12) nst = 6 rates = invgamma;
Subset13 = 3048–3831\3, 3834–4965\3	TIM + I+G:Subset13,	lset applyto = (13) nst = 6 rates = invgamma;
Subset14 = 12487–12551, 4966–5907\3	GTR + I+G:Subset14,	lset applyto = (14) nst = 6 rates = invgamma;
Subset15 = 4967–5907\3	K81UF + I+G:Subset15,	lset applyto = (15) nst = 6 rates = invgamma;
Subset16 = 7275–7560\3, 7563–8898\3, 4968–5907\3, 8901–10617\3	GTR + I+G:Subset16,	lset applyto = (16) nst = 6 rates = invgamma;
Subset17 = 5908–6921\3	GTR + I+G:Subset17,	lset applyto = (17) nst = 6 rates = invgamma;
Subset18 = 5909–6921\3	GTR + I+G:Subset18,	lset applyto = (18) nst = 6 rates = invgamma;
Subset19 = 6923–7272\3	TVM + I+G:Subset19,	lset applyto = (19) nst = 6 rates = invgamma;
Subset20 = 11205–11264, 7273–7560\3	TVM + G:Subset20,	lset applyto = (20) nst = 6 rates = gamma;
Subset21 = 7274–7560\3	K81UF + G:Subset21,	lset applyto = (21) nst = 6 rates = gamma;
Subset22 = 7561–8898\3, 11702–11759	TVM + I+G:Subset22,	lset applyto = (22) nst = 6 rates = invgamma;
Subset23 = 8900–10617\3, 7562–8898\3	GTR + I+G:Subset23,	lset applyto = (23) nst = 6 rates = invgamma;
Subset24 = 8899–10617\3	GTR + I+G:Subset24,	lset applyto = (24) nst = 6 rates = invgamma;
Subset25 = 10618–11139\3	GTR + I+G:Subset25,	lset applyto = (25) nst = 6 rates = invgamma;
Subset26 = 10619–11139\3	TVM + G:Subset26,	lset applyto = (26) nst = 6 rates = gamma;
Subset27 = 10620–11139\3	HKY + G:Subset27,	lset applyto = (27) nst = 2 rates = gamma;
Subset28 = 11140–11204, 11760–11825, 11454–11512	TVM + I+G:Subset28,	lset applyto = (28) nst = 6 rates = invgamma;
Subset29 = 12288–12351, 11265–11327	GTR + I+G:Subset29,	lset applyto = (29) nst = 6 rates = invgamma;
Subset30 = 11895–11958, 11328–11388	TVM + I+G:Subset30,	lset applyto = (30) nst = 6 rates = invgamma;
Subset31 = 11389–11453, 12024–12092	HKY + G:Subset31,	lset applyto = (31) nst = 2 rates = gamma;
Subset32 = 11513–11576	K81UF + I+G:Subset32,	lset applyto = (32) nst = 6 rates = invgamma;
Subset33 = 11631–11701, 11826–11894	TVM + I+G:Subset33,	lset applyto = (33) nst = 6 rates = invgamma;
Subset34 = 11959–12023, 12352–12420	TIM + G:Subset34,	lset applyto = (34) nst = 6 rates = gamma;
Subset35 = 12093–12154, 12223–12287	GTR + I+G:Subset35,	lset applyto = (35) nst = 6 rates = invgamma;
Subset36 = 12421–12486, 12155–12222	TVM + G:Subset36;	lset applyto = (36) nst = 6 rates = gamma;
C123TR-P1		
Subset1 = 3046–3831, 1–675	GTR + I+G:Subset1,	lset applyto = (1) nst = 6 rates = invgamma;
Subset2 = 676–831, 10618–11139	GTR + I+G:Subset2,	lset applyto = (2) nst = 6 rates = invgamma;
Subset3 = 832–2361	GTR + I+G:Subset3,	lset applyto = (3) nst = 6 rates = invgamma;
Subset4 = 2362–3045	GTR + I+G:Subset4,	lset applyto = (4) nst = 6 rates = invgamma;
Subset5 = 3832–4965	GTR + I+G:Subset5,	lset applyto = (5) nst = 6 rates = invgamma;
Subset6 = 11513–11576, 4966–5907	GTR + I+G:Subset6,	lset applyto = (6) nst = 6 rates = invgamma;
Subset7 = 5908–6921	TIM + I+G:Subset7,	lset applyto = (7) nst = 6 rates = invgamma;
Subset8 = 6922–7272	TIM + I+G:Subset8,	lset applyto = (8) nst = 6 rates = invgamma;
Subset9 = 7273–7560, 11205–11264	K81UF + G:Subset9,	lset applyto = (9) nst = 6 rates = gamma;
Subset10 = 7561–8898	GTR + I+G:Subset10,	lset applyto = (10) nst = 6 rates = invgamma;
Subset11 = 8899–10617	GTR + I+G:Subset11,	lset applyto = (11) nst = 6 rates = invgamma;

Subset12 = 11140–11204, 11454–11512, 11760–11825	TVM + I+G:Subset12,	lset applyto = (12) nst = 6 rates = invgamma;
Subset13 = 11265–11327, 12288–12351	GTR + I+G:Subset13,	lset applyto = (13) nst = 6 rates = invgamma;
Subset14 = 11328–11388, 11895–11958	TVM + I+G:Subset14,	lset applyto = (14) nst = 6 rates = invgamma;
Subset15 = 11389–11453, 12024–12092	HKY + G:Subset15,	lset applyto = (15) nst = 2 rates = gamma;
Subset16 = 11577–11630, 12487–12551	TVM + G:Subset16,	lset applyto = (16) nst = 6 rates = gamma;
Subset17 = 11631–11701, 11826–11894	TVM + I+G:Subset17,	lset applyto = (17) nst = 6 rates = invgamma;
Subset18 = 11702–11759, 13772–14528	GTR + G:Subset18,	lset applyto = (18) nst = 6 rates = gamma;
Subset19 = 11959–12023, 12352–12420	TIM + G:Subset19,	lset applyto = (19) nst = 6 rates = gamma;
Subset20 = 12093–12154, 12223–12287	GTR + I+G:Subset20,	lset applyto = (20) nst = 6 rates = invgamma;
Subset21 = 12155–12222, 12421–12486	TVM + G:Subset21,	lset applyto = (21) nst = 6 rates = gamma;
Subset22 = 12552–13771	GTR + I+G:Subset22;	lset applyto = (22) nst = 6 rates = invgamma;
C123TR-P2		
Subset1 = 1–675\3, 6922–7272\3	GTR + I+G:Subset1,	lset applyto = (1) nst = 6 rates = invgamma;
Subset2 = 2363–3045\3, 2–675\3	TVM + I+G:Subset2,	lset applyto = (2) nst = 6 rates = invgamma;
Subset3 = 10620–11139\3, 3–675\3, 6924–7272\3	TRN + I+G:Subset3,	lset applyto = (3) nst = 6 rates = invgamma;
Subset4 = 676–831\3	TIM + I+G:Subset4,	lset applyto = (4) nst = 6 rates = invgamma;
Subset5 = 677–831\3	GTR + G:Subset5,	lset applyto = (5) nst = 6 rates = gamma;
Subset6 = 678–831\3	GTR + G:Subset6,	lset applyto = (6) nst = 6 rates = gamma;
Subset7 = 832–2361\3	GTR + I+G:Subset7,	lset applyto = (7) nst = 6 rates = invgamma;
Subset8 = 833–2361\3	TVM + I+G:Subset8,	lset applyto = (8) nst = 6 rates = invgamma;
Subset9 = 834–2361\3	TIM + G:Subset9,	lset applyto = (9) nst = 6 rates = gamma;
Subset10 = 2362–3045\3, 11577–11630	GTR + I+G:Subset10,	lset applyto = (10) nst = 6 rates = invgamma;
Subset11 = 2364–3045\3	TRN + G:Subset11,	lset applyto = (11) nst = 6 rates = gamma;
Subset12 = 3832–4965\3, 3046–3831\3	GTR + I+G:Subset12,	lset applyto = (12) nst = 6 rates = invgamma;
Subset13 = 3047–3831\3, 3833–4965\3	GTR + I+G:Subset13,	lset applyto = (13) nst = 6 rates = invgamma;
Subset14 = 3048–3831\3, 3834–4965\3	TIM + I+G:Subset14,	lset applyto = (14) nst = 6 rates = invgamma;
Subset15 = 12487–12551, 4966–5907\3	GTR + I+G:Subset15,	lset applyto = (15) nst = 6 rates = invgamma;
Subset16 = 4967–5907\3	K81UF + I+G:Subset16,	lset applyto = (16) nst = 6 rates = invgamma;
Subset17 = 4968–5907\3, 8901–10617\3, 7275–7560\3, 7563–8898\3	GTR + I+G:Subset17,	lset applyto = (17) nst = 6 rates = invgamma;
Subset18 = 5908–6921\3	GTR + I+G:Subset18,	lset applyto = (18) nst = 6 rates = invgamma;
Subset19 = 5909–6921\3	GTR + I+G:Subset19,	lset applyto = (19) nst = 6 rates = invgamma;
Subset20 = 5910–6921\3	TIM + I+G:Subset20,	lset applyto = (20) nst = 6 rates = invgamma;
Subset21 = 6923–7272\3	TVM + I+G:Subset21,	lset applyto = (21) nst = 6 rates = invgamma;
Subset22 = 11205–11264, 7273–7560\3	TVM + G:Subset22,	lset applyto = (22) nst = 6 rates = gamma;
Subset23 = 7274–7560\3	K81UF + G:Subset23,	lset applyto = (23) nst = 6 rates = gamma;
Subset24 = 7561–8898\3	TVM + I+G:Subset24,	lset applyto = (24) nst = 6 rates = invgamma;
Subset25 = 7562–8898\3	GTR + I+G:Subset25,	lset applyto = (25) nst = 6 rates = invgamma;
Subset26 = 8899–10617\3	GTR + I+G:Subset26,	lset applyto = (26) nst = 6 rates = invgamma;
Subset27 = 8900–10617\3	GTR + I+G:Subset27,	lset applyto = (27) nst = 6 rates = invgamma;
Subset28 = 10618–11139\3	GTR + I+G:Subset28,	lset applyto = (28) nst = 6 rates = invgamma;
Subset29 = 10619–11139\3	GTR + I+G:Subset29,	lset applyto = (29) nst = 6 rates = invgamma;
Subset30 = 11140–11204, 11760–11825, 11454–11512	TVM + I+G:Subset30,	lset applyto = (30) nst = 6 rates = invgamma;
Subset31 = 11265–11327, 12288–12351	GTR + I+G:Subset31,	lset applyto = (31) nst = 6 rates = invgamma;
Subset32 = 11895–11958, 11328–11388	TVM + I+G:Subset32,	lset applyto = (32) nst = 6 rates = invgamma;
Subset33 = 12024–12092, 11389–11453	HKY + G:Subset33,	lset applyto = (33) nst = 2 rates = gamma;
Subset34 = 11513–11576, 12552–13771	GTR + I+G:Subset34,	lset applyto = (34) nst = 6 rates = invgamma;
Subset35 = 12155–12222, 11631–11701, 11826–11894	TVM + I+G:Subset35,	lset applyto = (35) nst = 6 rates = invgamma;
Subset36 = 12421–12486, 11702–11759	K81UF + G:Subset36,	lset applyto = (36) nst = 6 rates = gamma;
Subset37 = 11959–12023, 12352–12420	TIM + G:Subset37,	lset applyto = (37) nst = 6 rates = gamma;
Subset38 = 12093–12154, 12223–12287	GTR + I+G:Subset38,	lset applyto = (38) nst = 6 rates = invgamma;
Subset39 = 13772–14528	GTR + G:Subset39;	lset applyto = (39) nst = 6 rates = gamma;
C12-P2		

Subset1 = 1–450\2, 4615–4848\2	GTR + I+G:Subset1,	lset applyto = (1) nst = 6 rates = invgamma;
Subset2 = 1576–2030\2, 2–450\2	TVM + I+G:Subset2,	lset applyto = (2) nst = 6 rates = invgamma;
Subset3 = 451–554\2	TIM + I+G:Subset3,	lset applyto = (3) nst = 6 rates = invgamma;
Subset4 = 452–554\2	GTR + G:Subset4,	lset applyto = (4) nst = 6 rates = gamma;
Subset5 = 555–1574\2	GTR + I+G:Subset5,	lset applyto = (5) nst = 6 rates = invgamma;
Subset6 = 556–1574\2	TVM + I+G:Subset6,	lset applyto = (6) nst = 6 rates = invgamma;
Subset7 = 1575–2030\2	GTR + I+G:Subset7,	lset applyto = (7) nst = 6 rates = invgamma;
Subset8 = 2555–3310\2, 2031–2554\2	GTR + I+G:Subset8,	lset applyto = (8) nst = 6 rates = invgamma;
Subset9 = 2556–3310\2, 2032–2554\2	GTR + I+G:Subset9,	lset applyto = (9) nst = 6 rates = invgamma;
Subset10 = 3311–3938\2	GTR + I+G:Subset10,	lset applyto = (10) nst = 6 rates = invgamma;
Subset11 = 3312–3938\2	K81UF + I+G:Subset11,	lset applyto = (11) nst = 6 rates = invgamma;
Subset12 = 3939–4614\2	GTR + I+G:Subset12,	lset applyto = (12) nst = 6 rates = invgamma;
Subset13 = 3940–4614\2	GTR + I+G:Subset13,	lset applyto = (13) nst = 6 rates = invgamma;
Subset14 = 4616–4848\2	TVM + I+G:Subset14,	lset applyto = (14) nst = 6 rates = invgamma;
Subset15 = 4849–5040\2	TVM + G:Subset15,	lset applyto = (15) nst = 6 rates = gamma;
Subset16 = 4850–5040\2	K81UF + G:Subset16,	lset applyto = (16) nst = 6 rates = gamma;
Subset17 = 5041–5932\2	TVM + I+G:Subset17,	lset applyto = (17) nst = 6 rates = invgamma;
Subset18 = 5042–5932\2, 5934–7078\2	GTR + I+G:Subset18,	lset applyto = (18) nst = 6 rates = invgamma;
Subset19 = 5933–7078\2	GTR + I+G:Subset19,	lset applyto = (19) nst = 6 rates = invgamma;
Subset20 = 7079–7426\2	GTR + I+G:Subset20,	lset applyto = (20) nst = 6 rates = invgamma;
Subset21 = 7080–7426\2	TVM + I+G:Subset21;	lset applyto = (21) nst = 6 rates = invgamma;
C12R-P2		
Subset1 = 1–1220	GTR + I+G:Subset1,	lset applyto = (1) nst = 6 rates = invgamma;
Subset2 = 1221–1977	GTR + G:Subset2,	lset applyto = (2) nst = 6 rates = gamma;
Subset3 = 1978–2605\2	GTR + I+G:Subset3,	lset applyto = (3) nst = 6 rates = invgamma;
Subset4 = 1979–2605\2	K81UF + I+G:Subset4,	lset applyto = (4) nst = 6 rates = invgamma;
Subset5 = 2606–3281\2	GTR + I+G:Subset5,	lset applyto = (5) nst = 6 rates = invgamma;
Subset6 = 2607–3281\2	GTR + I+G:Subset6,	lset applyto = (6) nst = 6 rates = invgamma;
Subset7 = 3282–3737\2	GTR + I+G:Subset7,	lset applyto = (7) nst = 6 rates = invgamma;
Subset8 = 3283–3737\2, 8851–9299\2	TVM + I+G:Subset8,	lset applyto = (8) nst = 6 rates = invgamma;
Subset9 = 8850–9299\2, 3738–3971\2	GTR + I+G:Subset9,	lset applyto = (9) nst = 6 rates = invgamma;
Subset10 = 3739–3971\2	TVM + I+G:Subset10,	lset applyto = (10) nst = 6 rates = invgamma;
Subset11 = 7902–8657\2, 3972–4495\2	GTR + I+G:Subset11,	lset applyto = (11) nst = 6 rates = invgamma;
Subset12 = 7903–8657\2, 3973–4495\2	GTR + I+G:Subset12,	lset applyto = (12) nst = 6 rates = invgamma;
Subset13 = 4496–5387\2	TVM + I+G:Subset13,	lset applyto = (13) nst = 6 rates = invgamma;
Subset14 = 4497–5387\2, 5389–6533\2	GTR + I+G:Subset14,	lset applyto = (14) nst = 6 rates = invgamma;
Subset15 = 5388–6533\2	GTR + I+G:Subset15,	lset applyto = (15) nst = 6 rates = invgamma;
Subset16 = 6534–6881\2	GTR + I+G:Subset16,	lset applyto = (16) nst = 6 rates = invgamma;
Subset17 = 6535–6881\2	TVM + I+G:Subset17,	lset applyto = (17) nst = 6 rates = invgamma;
Subset18 = 6882–7901\2	GTR + I+G:Subset18,	lset applyto = (18) nst = 6 rates = invgamma;
Subset19 = 6883–7901\2	TVM + I+G:Subset19,	lset applyto = (19) nst = 6 rates = invgamma;
Subset20 = 8658–8849\2	TVM + G:Subset20,	lset applyto = (20) nst = 6 rates = gamma;
Subset21 = 8659–8849\2	K81UF + G:Subset21,	lset applyto = (21) nst = 6 rates = gamma;
Subset22 = 9300–9403\2	TIM + I+G:Subset22,	lset applyto = (22) nst = 6 rates = invgamma;
Subset23 = 9301–9403\2	GTR + G:Subset23;	lset applyto = (23) nst = 6 rates = gamma;
C12T-P2		
Subset1 = 8774–8838, 1–628\2, 7427–7491	GTR + I+G:Subset1,	lset applyto = (1) nst = 6 rates = invgamma;
Subset2 = 2–628\2	K81UF + I+G:Subset2,	lset applyto = (2) nst = 6 rates = invgamma;
Subset3 = 629–1304\2	GTR + I+G:Subset3,	lset applyto = (3) nst = 6 rates = invgamma;
Subset4 = 630–1304\2	GTR + I+G:Subset4,	lset applyto = (4) nst = 6 rates = invgamma;
Subset5 = 7864–7917, 1305–1760\2	GTR + I+G:Subset5,	lset applyto = (5) nst = 6 rates = invgamma;
Subset6 = 6874–7322\2, 1306–1760\2	TVM + I+G:Subset6,	lset applyto = (6) nst = 6 rates = invgamma;
Subset7 = 6873–7322\2, 1761–1994\2	GTR + I+G:Subset7,	lset applyto = (7) nst = 6 rates = invgamma;
Subset8 = 1762–1994\2	TVM + I+G:Subset8,	lset applyto = (8) nst = 6 rates = invgamma;
Subset9 = 5925–6680\2, 1995–2518\2	GTR + I+G:Subset9,	lset applyto = (9) nst = 6 rates = invgamma;
Subset10 = 1996–2518\2, 5926–6680\2	GTR + I+G:Subset10,	lset applyto = (10) nst = 6 rates = invgamma;
Subset11 = 2519–3410\2, 7989–8046	TVM + I+G:Subset11,	lset applyto = (11) nst = 6 rates = invgamma;

Subset12 = 2520–3410\2, 3412–4556\2	GTR + I+G:Subset12,	lset applyto = (12) nst = 6 rates = invgamma;
Subset13 = 3411–4556\2	GTR + I+G:Subset13,	lset applyto = (13) nst = 6 rates = invgamma;
Subset14 = 4557–4904\2	GTR + I+G:Subset14,	lset applyto = (14) nst = 6 rates = invgamma;
Subset15 = 4558–4904\2	TVM + I+G:Subset15,	lset applyto = (15) nst = 6 rates = invgamma;
Subset16 = 4905–5924\2	GTR + I+G:Subset16,	lset applyto = (16) nst = 6 rates = invgamma;
Subset17 = 4906–5924\2	TVM + I+G:Subset17,	lset applyto = (17) nst = 6 rates = invgamma;
Subset18 = 6681–6872\2, 7492–7551	TVM + G:Subset18,	lset applyto = (18) nst = 6 rates = gamma;
Subset19 = 6682–6872\2	K81UF + G:Subset19,	lset applyto = (19) nst = 6 rates = gamma;
Subset20 = 7323–7426\2	TIM + I+G:Subset20,	lset applyto = (20) nst = 6 rates = invgamma;
Subset21 = 7324–7426\2, 7918–7988, 8113–8181	GTR + I+G:Subset21,	lset applyto = (21) nst = 6 rates = invgamma;
Subset22 = 7800–7863, 7552–7614	HKY + I+G:Subset22,	lset applyto = (22) nst = 2 rates = invgamma;
Subset23 = 8182–8245, 7615–7675	TVM + I+G:Subset23,	lset applyto = (23) nst = 6 rates = invgamma;
Subset24 = 7676–7740, 8311–8379	HKY + G:Subset24,	lset applyto = (24) nst = 2 rates = gamma;
Subset25 = 8047–8112, 7741–7799	TVM + I+G:Subset25,	lset applyto = (25) nst = 6 rates = invgamma;
Subset26 = 8639–8707, 8246–8310	TIM + G:Subset26,	lset applyto = (26) nst = 6 rates = gamma;
Subset27 = 8380–8441, 8510–8574	GTR + I+G:Subset27,	lset applyto = (27) nst = 6 rates = invgamma;
Subset28 = 8442–8509, 8708–8773	TVM + G:Subset28,	lset applyto = (28) nst = 6 rates = gamma;
Subset29 = 8575–8638	TIM + I+G:Subset29;	lset applyto = (29) nst = 6 rates = invgamma;
C12TR-P2		
Subset1 = 1–628\2, 8774–8838	GTR + I+G:Subset1,	lset applyto = (1) nst = 6 rates = invgamma;
Subset2 = 2–628\2	K81UF + I+G:Subset2,	lset applyto = (2) nst = 6 rates = invgamma;
Subset3 = 629–1304\2	GTR + I+G:Subset3,	lset applyto = (3) nst = 6 rates = invgamma;
Subset4 = 630–1304\2	GTR + I+G:Subset4,	lset applyto = (4) nst = 6 rates = invgamma;
Subset5 = 7864–7917, 1305–1760\2	GTR + I+G:Subset5,	lset applyto = (5) nst = 6 rates = invgamma;
Subset6 = 6874–7322\2, 1306–1760\2	TVM + I+G:Subset6,	lset applyto = (6) nst = 6 rates = invgamma;
Subset7 = 6873–7322\2, 1761–1994\2	GTR + I+G:Subset7,	lset applyto = (7) nst = 6 rates = invgamma;
Subset8 = 1762–1994\2	TVM + I+G:Subset8,	lset applyto = (8) nst = 6 rates = invgamma;
Subset9 = 5925–6680\2, 1995–2518\2	GTR + I+G:Subset9,	lset applyto = (9) nst = 6 rates = invgamma;
Subset10 = 5926–6680\2, 1996–2518\2	GTR + I+G:Subset10,	lset applyto = (10) nst = 6 rates = invgamma;
Subset11 = 2519–3410\2	TVM + I+G:Subset11,	lset applyto = (11) nst = 6 rates = invgamma;
Subset12 = 2520–3410\2, 3412–4556\2	GTR + I+G:Subset12,	lset applyto = (12) nst = 6 rates = invgamma;
Subset13 = 3411–4556\2	GTR + I+G:Subset13,	lset applyto = (13) nst = 6 rates = invgamma;
Subset14 = 4557–4904\2	GTR + I+G:Subset14,	lset applyto = (14) nst = 6 rates = invgamma;
Subset15 = 4558–4904\2	GTR + I+G:Subset15,	lset applyto = (15) nst = 6 rates = invgamma;
Subset16 = 4905–5924\2	GTR + I+G:Subset16,	lset applyto = (16) nst = 6 rates = invgamma;
Subset17 = 4906–5924\2	TVM + I+G:Subset17,	lset applyto = (17) nst = 6 rates = invgamma;
Subset18 = 6681–6872\2, 7492–7551	TVM + G:Subset18,	lset applyto = (18) nst = 6 rates = gamma;
Subset19 = 6682–6872\2	K81UF + G:Subset19,	lset applyto = (19) nst = 6 rates = gamma;
Subset20 = 7323–7426\2	TIM + I+G:Subset20,	lset applyto = (20) nst = 6 rates = invgamma;
Subset21 = 7324–7426\2	GTR + G:Subset21,	lset applyto = (21) nst = 6 rates = gamma;
Subset22 = 7427–7491, 8047–8112, 7741–7799	TVM + I+G:Subset22,	lset applyto = (22) nst = 6 rates = invgamma;
Subset23 = 7800–7863, 7552–7614	HKY + I+G:Subset23,	lset applyto = (23) nst = 2 rates = invgamma;
Subset24 = 8182–8245, 7615–7675	TVM + I+G:Subset24,	lset applyto = (24) nst = 6 rates = invgamma;
Subset25 = 8311–8379, 7676–7740	HKY + G:Subset25,	lset applyto = (25) nst = 2 rates = gamma;
Subset26 = 8442–8509, 8113–8181, 7918–7988	TVM + I+G:Subset26,	lset applyto = (26) nst = 6 rates = invgamma;
Subset27 = 8708–8773, 7989–8046	K81UF + G:Subset27,	lset applyto = (27) nst = 6 rates = gamma;
Subset28 = 8639–8707, 8246–8310	TIM + G:Subset28,	lset applyto = (28) nst = 6 rates = gamma;
Subset29 = 8510–8574, 8380–8441	GTR + I+G:Subset29,	lset applyto = (29) nst = 6 rates = invgamma;
Subset30 = 8575–8638	TRN + I+G:Subset30,	lset applyto = (30) nst = 6 rates = invgamma;
Subset31 = 8839–10058	GTR + I+G:Subset31,	lset applyto = (31) nst = 6 rates = invgamma;
Subset32 = 10059–10815	GTR + G:Subset32;	lset applyto = (32) nst = 6 rates = gamma;
